# Regulation of Epithelial Sodium Transport by SARS-CoV-2 Is Closely Related with Fibrinolytic System-Associated Proteins

**DOI:** 10.3390/biom13040578

**Published:** 2023-03-23

**Authors:** Tingyu Wang, Yiman Zhai, Hao Xue, Wei Zhou, Yan Ding, Hongguang Nie

**Affiliations:** Department of Stem Cells and Regenerative Medicine, College of Basic Medical Science, China Medical University, Shenyang 110122, China

**Keywords:** SARS-CoV-2, epithelial sodium channel, plasmin, acute respiratory distress syndrome, furin site

## Abstract

Dyspnea and progressive hypoxemia are the main clinical features of patients with coronavirus disease 2019 (COVID-19), which is caused by severe acute respiratory syndrome coronavirus 2 (SARS-CoV-2). Pulmonary pathology shows diffuse alveolar damage with edema, hemorrhage, and the deposition of fibrinogens in the alveolar space, which are consistent with the Berlin Acute Respiratory Distress Syndrome Criteria. The epithelial sodium channel (ENaC) is a key channel protein in alveolar ion transport and the rate-limiting step for pulmonary edema fluid clearance, the dysregulation of which is associated with acute lung injury/acute respiratory distress syndrome. The main protein of the fibrinolysis system, plasmin, can bind to the furin site of γ-ENaC and induce it to an activation state, facilitating pulmonary fluid reabsorption. Intriguingly, the unique feature of SARS-CoV-2 from other β-coronaviruses is that the spike protein of the former has the same furin site (RRAR) with ENaC, suggesting that a potential competition exists between SARS-CoV-2 and ENaC for the cleavage by plasmin. Extensive pulmonary microthrombosis caused by disorders of the coagulation and fibrinolysis system has also been seen in COVID-19 patients. To some extent, high plasmin (ogen) is a common risk factor for SARS-CoV-2 infection since an increased cleavage by plasmin accelerates virus invasion. This review elaborates on the closely related relationship between SARS-CoV-2 and ENaC for fibrinolysis system-related proteins, aiming to clarify the regulation of ENaC under SARS-CoV-2 infection and provide a novel reference for the treatment of COVID-19 from the view of sodium transport regulation in the lung epithelium.

## 1. Introduction

Ever since the coronavirus disease 2019 (COVID-19) was first reported in Wuhan, it has become a worldwide pandemic for more than 3 years. Although most people are now asymptomatic or mildly infected, the number of COVID-19 deaths is still increasing. In addition, more and more variants of the virus with unknown pathogenesis remain a potential threat to human health. As the pathogen causing COVID-19, severe acute respiratory syndrome coronavirus 2 (SARS-CoV-2) is highly infectious and fast spreading, with the characteristic of appearing round or oval in shape and 60–140 nm in diameter. The surface of the virus consists of four structural proteins, named spike protein, membrane protein, envelope protein, and nucleocapsid protein, respectively [1]. SARS-CoV-2 shares about a 76% amino acid sequence in the spike protein with SARS-CoV, another β-coronavirus, both of which can participate in the viral membrane fusion mediated by transmembrane serine protease 2 (TMPRSS2) [2,3]. The unique feature of SARS-CoV-2 is the insertion of four amino acid residues at the junction of S1 and S2 subunits of the spike protein, which creates a multi-base furin site (RRAR) that can be cleaved by furin and other proteases, including plasmin, thereby accelerating the invasion of the virus into host cells [4,5,6]. During the pandemic, the Alpha, Beta, Gamma, and Delta variants of SARS-CoV-2 all carried the D614G mutation, which indirectly enhanced the cleavability of the furin site compared to the original Wuhan strain [7]. The main reason that the Delta variant has replaced the Alpha one as a popular strain is that the P681R, other than the P681H mutation, arises from the same amino acid site and adds a powerful basic amino acid arginine at the S1/S2 site in the Delta variant but not Alpha one, which enhances the cleavage of the site and leads to increased cell surface-mediated viral invasion [8,9].

Under SARS-CoV-2 infection, extensive pulmonary microthrombosis has been seen by the disorder of coagulation and the fibrinolysis system. High plasmin (ogen) is a common risk factor for SARS-CoV-2 infection, for the increased cleavage by plasmin at 682RRAR/S686 of the spike protein, which accelerates virus invasion [10]. Thus COVID-19 can be characterized as both a respiratory and systemic thrombotic disease, traditionally known as COVID-19-associated coagulopathy [11,12]. Coincidentally, the epithelial sodium channel (ENaC), a key player in the regulation of water and salt homeostasis in vivo, shares the same furin site, which plays an important role in ENaC activation. It has been suggested that SARS-CoV-2 may interfere with pulmonary water-salt balance by competing with the plasmin cleavage mechanism of ENaC, which can also explain why SARS-CoV-2 causes the downregulation of ENaC and pulmonary edema appears in COVID-19 patients [13,14].

A recent study has shown that the knockdown of furin using CRISPR-Cas9 technology reduces but does not completely inhibit the production of infectious SARS-CoV-2 viral particles in 293T cells, suggesting that furin is the protein that primarily, but may not essentially, promote SARS-CoV-2 invasion [15]. During acute responses such as viral infections, the regulation of ENaC is closely related to fibrinolytic system-associated proteins through specific signaling pathways involving protein expression, ubiquitination degradation, cellular sublocalization, channel opening probability, etc. [16,17].

## 2. Relationship of SARS-CoV-2 Infection and Lung Impairment

Acute lung injury/acute respiratory distress syndrome (ALI/ARDS) is a common critical illness that can be caused by direct and indirect diffuse impairment to the lung parenchyma from a variety of causes, such as pneumonia and sepsis, with a high morbidity and mortality rate [18,19]. The main pathological features of ALI/ARDS are inflammation, pulmonary edema, transparent membrane formation, the destruction of the alveolar–capillary barrier, and increased protein permeability with bilateral chest imaging opacity [20]. Critically ill patients present with respiratory failure, septic shock, or multi-organ dysfunction and usually require invasive mechanical ventilation [21]. As one of the important pathological features of ALI/ARDS, pulmonary edema is often accompanied by impaired alveolar fluid clearance. According to the analysis of clinical features and pulmonary pathological changes, the occurrence of hypoxemia in COVID-19 is related to pulmonary edema caused by viral infection [22]. Mammalian ENaC mainly consists of three homologous subunits α, β, and γ, and is primarily localized on the apical side of alveolar epithelial cells, the downregulation of which may lead to the development of ALI/ARDS [23,24,25,26,27].

### 2.1. ARDS Different from ‘Classic’ Occurs in COVID-19 Patients

An analysis of the autopsy and biopsy results from 662 patients in 58 articles revealed histopathology in COVID-19 patients and showed pulmonary edema, marked exudative and proliferative responses, and inflammatory infiltrates [28]. The clinical severity of COVID-19 patients varies widely and is classified by the National Health Association into five categories: asymptomatic, mild, moderate, severe, and critical. The majority of patients develop asymptomatic, mild, or moderate disease and are usually treated at home without oxygen therapy [29,30], whereas patients with severe/critical disease are characterized by arterial hypoxemia and respiratory distress, which seem similar to patients with ‘classic’ ARDS [31]. However, some studies showed that both pulmonary vascular permeability and extravascular lung water indexes were elevated to a higher degree in ARDS due to SARS-CoV-2 infection compared with ‘classic’ ARDS, reflecting the higher severity of fluid accumulation in COVID-19 patients [32,33,34]. The pulmonary edema observed in COVID-19 patients indicates how water homeostasis is destroyed, which is mostly related to the imbalance of ion transport and, more specifically, impaired sodium reabsorption in the lung epithelium [14].

### 2.2. SARS-CoV-2 Promotes ENaC Degradation

**Figure 1:**
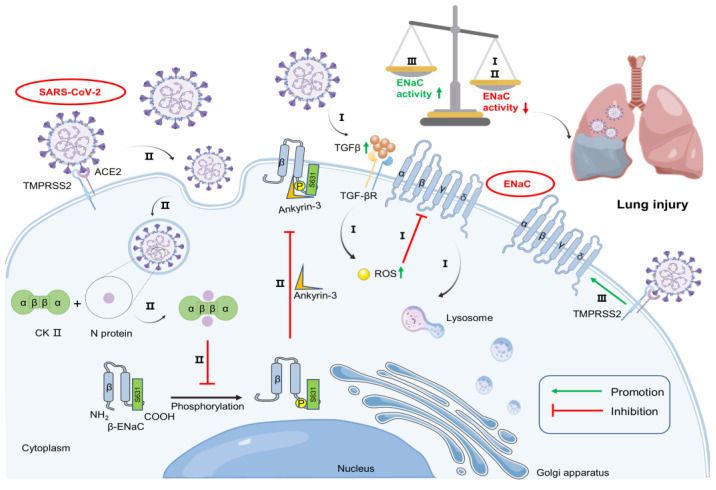
Mechanism diagram of epithelial sodium channel (ENaC) regulation in vivo during severe acute respiratory syndrome coronavirus 2 (SARS-CoV-2) invasion. (**I**): Elevated transforming growth factor-beta (TGF-β) binds to receptor (TGF-βR), causing increased reactive oxygen species (ROS) and leads to the internalization of ENaC. (**II**): SARS-CoV-2 envelope (N) protein interacts with casein kinase II (CK II) and interferes with its ability to phosphorylate and localize β-ENaC to the apical membrane side. (**III**): Transmembrane serine protease 2 (TMPRSS2) interacts with SARS-CoV-2 spike (S) protein, resulting in the diminished inhibition of ENaC. The sum effect reduces ENaC activity and leads to lung injury.

Transforming growth factor-β (TGF-β), a crucial factor in ARDS pathophysiology, is expressed significantly high in the lung tissues of COVID-19 patients, which activates phospholipase D1, phosphatidylinositol-4-phosphate 5-kinase 1α and NADPH oxidase 4 to generate reactive oxygen species (ROS). Although opinions on the regulation of ENaC by ROS are divergent, the dominant one is that ROS can drive the internalization of ENaC [35,36,37,38]. Elevated ROS may activate mitogen-activated protein kinases and strengthen the extracellular signal-regulated kinase 1/2 phosphorylation, which promotes the Nedd4-2-mediated ubiquitinated degradation of ENaC and negatively regulates ENaC, blocking sodium ion transport and causing persistent pulmonary edema accordingly (Figure 1I) [38,39,40]. As expected, GLPG-0187, as an inhibitor of TGF-β, can block SARS-CoV-2 pseudovirus infection in airway epithelial cells, thereby reducing disease severity [41].

### 2.3. SARS-CoV-2 May Change the Subcellular Localization of ENaC

Subcellular localization means that mature proteins must be inside specific sub-cells to perform stable biological functions. Common sub-cells are the nucleus, cell membrane, endoplasmic reticulum, Golgi apparatus, cytoplasm, and mitochondria. The subcellular localization of ENaC on the apical side of epithelial cells determines either competition for the furin site by the SARS-CoV-2 spike protein in the Golgi apparatus or the failure of cleaved one to be smoothly transported to the apical side, which results in the functional loss of ENaC in pulmonary fluid regulation. It has been demonstrated that by interfering with casein kinase II (CK II) signaling or knocking down CK II phosphorylation and ankyrin-3 binding sites, the translocation and activity of ENaC are reduced, proving that CK II is essential for the function of ENaC and proper localization to the apical side [42,43,44]. A recent quantitative mass spectrometry-based phosphorylation proteomics study showed that two subunits of CK II, CSNK2B, and CSNK2A2, interacted with SARS-CoV-2 N proteins to drive changes by affecting the subcellular localization of CK II or sterically blocking the entry of kinases, which may interfere with the phosphorylation of β-ENaC and its subcellular localization (Figure 1II) [45].

### 2.4. SARS-CoV-2 Relieves the Inhibitory Effect of TMPRSS2 on ENaC Expression

When the effect of TMPRSS2 on ENaC was initially investigated, the co-expression of human TMPRSS2 with rat ENaC in Xenopus oocytes significantly reduced the ENaC-mediated current and protein expression of ENaC, which was thought to be due to TMPRSS2 enhancing the proteolytic degradation of ENaC [46]. The expression of TMPRSS2 on the cell surface was regulated by a guanine-rich sequence in the promoter region, which is capable of forming an intracellular potassium ion-stabilized G-quadruplex structure [47]. During the SARS-CoV-2 invasion of host cells, the overstimulation of the ACE/Ang II/AT1R axis causes the loss of intracellular potassium ions and the increased expression of TMPRSS2 as a result [48]. In an inhibitory feedback loop, TMPRSS2 reduces ENaC activity, whereas, in the case of COVID-19, the involvement of TMPRSS2 in the invasion process by cleaving the S2’ site of the viral spike protein may attenuate its inhibitory effect on ENaC [46,49,50]. Intriguingly, conflicting data have been reported on the effect of TMPRSS2 on ENaC activity under normal conditions. TMPRSS2 was reported to hydrolytically activate ENaC by cleaving the γ-subunit of the channel at multiple sites, which was largely dependent on the catalytic activity of TMPRSS2 but not necessarily on its activity at the cell surface [49]. Considering that the proteolytic ENaC activation by TMPRSS2 may occur intracellularly before the channel matures, viral hijacking of TMPRSS2 at the plasma membrane could block the enhanced degradation of ENaC, whose activity could be increased eventually under SARS-CoV-2 infection (Figure 1III) [50].

### 2.5. Pro-Inflammatory Mediators Influence ENaC Expression under SARS-CoV-2 Infection

During SARS-CoV-2 infection, a cytokine storm occurred and was characterized by the high-level activation of immune cells and excessive production of massive pro-inflammatory mediators, which may explain the development of severe ARDS and is the main cause of disease severity and death in COVID-19 patients [51,52,53]. In a study of mouse macrophage migration, interferons (IFNs) and tumor necrosis factor-alpha (TNF-α) were found to reduce the whole cell α-ENaC protein by more than 50% [52]. Of note, increased mRNA expression of IFN-λ was observed in bronchoalveolar fluid and nasopharyngeal samples from COVID-19 patients, which impaired lung epithelial cell proliferation, differentiation, and repair, caused lung injury and susceptibility to overlap with bacterial infections [54]. TNF-α is an innate immune cytokine that presents during lung infection and has a profound influence on the ability of alveolar epithelial cells to transport sodium ions. It has been shown that TNF-α reduced the mRNA expression of α, β and γ-ENaC and halved the protein expression of α-ENaC in alveolar epithelial cells. Furthermore, there was a strong correlation between the reduction in amiloride-sensitive currents and α-ENaC mRNA expression at different TNF-α concentrations [55]. Similarly, interleukin (IL)-1β reduced the expression of α and β-ENaC via p38 MAPK [53].

## 3. Dysfunction of Coagulation and Fibrinolysis System Caused by SARS-CoV-2 Infection

Under normal conditions, the fibrinogen system maintains fibrin dynamic homeostasis through fibrin degradation by the tissue factor and fibrin formation of protein C pathways, respectively [56]. When the fibrinolysis system is stimulated, the tissue-type plasminogen activator (t-PA) and urokinase plasminogen activator (uPA) hydrolyse the Arg560–Val561 peptide bond of plasminogen and convert it into plasmin, thereby degrading fibrin. During acute viral infection, the upregulation of the fibrinolysis system leads to fibrinolysis, as well as producing the plasminogen activator inhibitor-1 (PAI-1), which then inhibits the plasminogen activator and leads to microvascular thrombosis.

**Figure 2:**
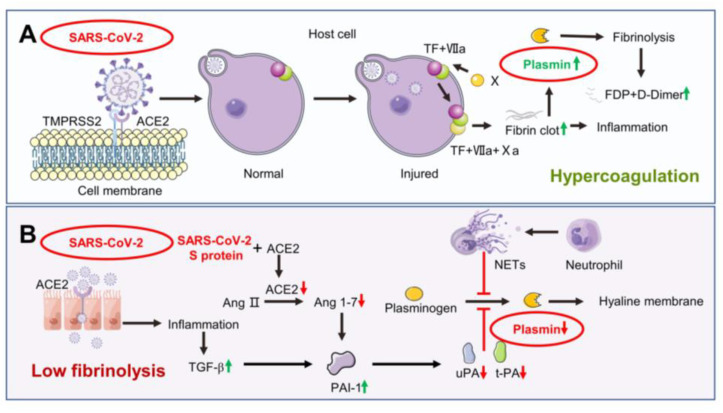
Possible process of coagulation and fibrinolysis in vivo during severe acute respiratory syndrome coronavirus 2 (SARS-CoV-2) infection. (**A**) SARS-CoV-2 spike (S) protein binds to angiotensin-converting enzyme 2 (ACE2) receptors and facilitates virus invasion into host cells with the assistance of type II transmembrane serine protease (TMPRSS2). Injured host cells activate the coagulation cascade through TF-VIIa (tissue factor pathway) to promote fibrin production (hypercoagulable state). Overactivation of the fibrinolysis system results in increased plasmin production. Elevated plasmin degrades fibrin, leading to high D-Dimer levels and triggers a series of inflammatory reactions. (**B**) Activation of neutrophils and neutrophil elastase-associated DNA (NE-DNA) complexes formed by neutrophil extracellular traps (NETs) degrade plasminogen and inhibit its activation to plasmin. ACE2 receptor is internalized after binding to the SARS-CoV-2 S protein and fails to decompose angiotensin II (Ang II) into angiotensin1-7 (Ang1-7). Meanwhile, inflammatory reactions lead to increased transforming growth factor-β (TGF-β) levels. Both Ang1-7 decrease and TGF-β elevation increases plasminogen activator inhibitor-1 (PAI-1), thereby inhibiting urokinase/tissue-type plasminogen activator (uPA/t-PA) and plasminogen to plasmin (low fibrinolysis state), leading to the formation of a hyaline membrane.

In autopsy specimens from COVID-19 patients, in addition to diffuse alveolar damage [57], peri-alveolar microthrombosis and intra-alveolar hemorrhage can be observed [58]. Accordingly, COVID-19 can be characterized as both a respiratory and systemic thrombotic disease, traditionally known as COVID-19-associated coagulopathy [59]. The clinical course is accompanied by a hypercoagulable state, which initially involves the pulmonary microvascular system, with progressive systemic involvement leading to distant organ thrombosis and multi-organ dysfunction syndrome, indicating a high risk of microthrombotic and macrothrombotic embolism [60,61]. The hyperactivation of the coagulation system leads to the increased production of plasmin, the degradation of fibrin, and significantly increased D-dimer and fibrin (ogen) degradation product values, which might be associated with adverse outcomes (Figure 2A) [62]. Hypercoagulation and the inhibition of fibrinolysis in the alveoli are key pathophysiological characteristics and important causes of high mortality in ARDS [63], which can lead to pulmonary vascular microthrombosis, alveolar fibrin deposition, V/Q ratio imbalance, pulmonary fluid accumulation, reduced pulmonary compliance, diffusion disorder, refractory hypoxemia, and pulmonary fibrosis [64,65]. Hence, clarifying the mechanism and process of the coagulation and fibrinolysis system in vivo under SARS-CoV-2 infection can help target and alleviate the adverse progression of pulmonary edema and diffuse alveolar injury in COVID-19 patients to achieve the effect of intervention treatment.

The fibrinolysis balance in the lung is dynamic in COVID-19. When viruses invade host cells in the early stages of the disease, uPA/uPAR, plasmin (ogen), and t-PA tilt the balance toward a high fibrinolysis state. Raised plasmin increases the degradation of fibrin in the alveoli, producing more D-dimers and increasing the risk of alveolar hemorrhage. SARS-CoV-2 induced TGF-β increase and the p53 signaling pathway activation then conjugate sand inhibits uPA/uPAR mRNA expression while enhancing PAI-1 mRNA levels and swinging the balance towards a low fibrinolysis state (Figure 2B) [66,67,68].

## 4. Regulation of Epithelial Sodium Transport by SARS-CoV-2 Related with Plasmin-Associated Proteins

Plasmin is formed by the NH_2_-terminal heavy chain and COOH-terminal light chain, linked by 2 disulfide bonds [69]. Elevated plasmin is a common feature in patients with underlying conditions (including hypertension, diabetes, cardiovascular disease, cerebrovascular disease, and chronic kidney disease) who are susceptible to COVID-19 [12]. The occurrence of fibrinolytic inactivation was found in critically ill COVID-19 patients and was characteristic of venous thromboembolism [70,71,72]. SARS-CoV-2 damages the endoplasmic reticulum and promotes the activation of the coagulation cascade through the up-regulation of tissue factor and down-regulation of protein C in ALI/ARDS [56].

### 4.1. Plasmin Participates in the Proteolytic Cleavage of ENaC

The activation of ENaC was achieved by the proteolytic cleavage to remove the inhibitory fragments embedded in the α and γ subunits and increased the channel opening probability [24,73]. The two furin sites in the α subunit were cleaved by the furin protein twice in the Golgi to release the inhibitory fragment of 26 amino acid residues, then ENaC was transported to the apical side, transforming the channel into a moderately activated state [74]. In contrast, for the γ subunit, furin could only cleave once [75]. In a lot of studies there, increased ENaC activity was observed in response to the applied proteases, implying that ENaC activity can be further increased by the proteases. This aligns with the concept that ENaC open probability relies on proteolytic cleavage. In recent years, besides plasmin, caspase-3, trypsin, neutrophil elastase, kallikrein, and uPA have been reported to be able to perform the secondary cleavage of γ subunits, converting ENaC to a high activity state [76,77,78].

### 4.2. Plasmin Directly Regulates the Opening Probability of ENaC

As an efficient serine protease, plasmin shares cleavage sites with trypsin I and IV, uPA, elastase, chymotrypsin, and furin. Zhao R. et al. demonstrated that plasmin enhanced pulmonary fluid clearance in human and injured mouse lungs in vitro, and seven cleavage sites in the human γ-ENaC protein were identified for plasmin to increase the probability of the ENaC opening [79]. Based on the diffuse alveolar injury in COVID-19 patients and the adverse clinical outcome of ARDS, it is suggested that ENaC activity is decreased under SARS-CoV-2 infection [13,17,80]. The reason is presumed to be that the SARS-CoV-2 spike protein has a more preferred plasmin cleavage site than γ-ENaC, just as different proteases have different preferences for certain cleavage sites [81].

Depending on the route of viral invasion into host cells, SARS-CoV-2 and ENaC can undergo two competition events for plasmin at different spatial and temporal levels. The spike protein is first cleaved at the S1/S2 furin site by membrane proteins or exocytotic proteins (e.g., plasmin) to induce conformational changes in the S1 subunit and bind to host cell ACE2 receptors, exposing the S2’ cleavage site for the subsequent membrane fusion process. Therefore, the first competition for plasmin occurs between SARS-CoV-2 and incompletely cleaves γ-ENaC at the cell membrane, which may lead to a decrease in ENaC channel activity. After the new viral particles assembled, intracellular proteins such as plasmin or furin proteases in Golgi pre-activate the newly assembled SARS-CoV-2 by binding to the S1/S2 furin site, which may perform the cleavage of the α subunit another time and the second competition between SARS-CoV-2 and α-ENaC would occur [17,82].

### 4.3. Angiotensin II Reduces Plasmin Expression to Regulate ENaC Activity

The renin-angiotensin system consists of a series of enzymatic reactions that generate angiotensin II (Ang II) from angiotensinogen, which is an important effector molecule, and its physiological importance as a vasoconstrictor is well known. It has also been shown that Ang II is a major regulator of fibrinolysis function homeostasis [83]. The dysregulation of the renin-angiotensin system is associated with the development of ALI/ARDS in patients with COVID-19 [84]. Under SARS-CoV-2 infection, the activity of angiotensin-converting enzyme 2 is decreased for host cell membrane binding to the viral spike protein, which leads to the reduction in Ang II degradation to angiotensin 1-7 (Ang 1-7) (Figure 2B) [85,86]. The decreased Ang 1-7 leads to the elevation of PAI-1 expression and the inhibition of t-PA/uPA, which decreases plasmin production accordingly [87,88]. The accumulation of Ang II combines with the pulmonary cell receptor angiotensin II type 1a receptor and promotes the excessive activation of ENaC, causing potassium ion loss, even hypokalemia, which collapses cellular osmotic pressure and leads to the swelling of the lung and rupture or necrosis of the cells [48,89,90].

### 4.4. Neutrophil Elastase May Inhibit Plasmin Expression to Regulate ENaC Activity

Neutrophils are closely associated with the development of ARDS, and the neutrophil extracellular traps (NETs) released by SARS-CoV-2 activated neutrophils promote lung epithelial cell death in vitro [91,92]. Patients with severe COVID-19 develop ARDS and may progress to cytokine storm syndrome, which recruits excessive cytokines and immune cells to the lung, resulting in diffuse alveolar injury, organ dysfunction, and even death [93]. NETs are the second bactericidal mechanism for neutrophils, consisting of DNA and granule proteins. The former is the main part of NETs and forms a backbone structure that holds various protein particles in place, and the latter consists of NE, histone G, myeloperoxidase, etc. Cruz B et al. demonstrated that NETs containing active NE–DNA complexes reduce plasminogen to fragments and enhance PAI-1 activity, thereby preventing plasminogen formation by decreasing local plasminogen concentrations and lessening plasmin, potentially inhibiting ENaC activation indirectly [94].

## 5. Emerging SARS-CoV-2 Therapeutics Associated with Fibrinolytic System Regulation of ENaC

Although the successful launch of the SARS-CoV-2 vaccine has somewhat curbed the spread of the virus, a scientific and sustainable therapeutic approach is needed to truly end the COVID-19 pandemic. At present, drugs for the treatment of SARS-CoV-2 infection are still being developed, among which the action mechanism associated with regulated ENaC activity and expression in some emerging pharmaceutical agents may achieve a promising therapeutic effect.

### 5.1. Soluble ACE2

Based on the mechanism of SARS-CoV-2 invasion, a modified form of soluble ACE2, called human recombinant soluble ACE2 (hrsACE2), that competitively binds to the virus would be beneficial for the treatment of COVID-19 [95]. As a promising drug for the treatment of COVID-19, hrsACE2 has an attractive potential and a good scientific basis. The binding of hrsACE2 to the spike protein of the virus may mediate SARS-CoV-2 neutralization and rescue cellular ACE2 activity, which is related to the protection of multiple organs from ENaC dysregulation by reducing Ang II levels [96]. As Ang 1-7 increases, the interaction with the Mas-related G protein-coupled receptor downregulates PAI-1, which makes the increase in plasmin activity and the normalization of ENaC activity feasible [97]. In in vitro cell experiments and engineered organoids, it was found that hrsACE2 could effectively reduce the viral load and neutralize SARS-CoV-2 in the early stage of infection [98]. In phase 1 and phase 2 clinical studies, the safety performance of hrsACE2 was within the acceptable range [99].

### 5.2. Protease Inhibitors

Inhibitors of proteases such as TMPRSS2 that mediate SARS-CoV-2 invasion are also targets for novel therapeutic agents [100]. As serine protease inhibitors, camostat mesylate and nafamostat mesylate can exert antiviral effects and enhance ENaC activity by blocking TMPRSS2 under SARS-CoV-2 infection [101]. Meanwhile, plasmin activity can also be reduced, which may impair ENaC function and affect their clinical application to some extent in the context of SARS-CoV-2 infection. Compared with camostat mesylate, the greater efficiency of nafamostat mesylate in blocking SARS-CoV-2 infection in human lung cells has led to clinical trials in combination with antivirals for the treatment of COVID-19 [102]. However, one of the adverse events that nafamostat mesylate produces is hyperkaliemia due to ENaC inhibition, which may worsen respiratory disease in clinical applications [103].

Another TMPRSS2 inhibitor with broad distribution capability, bromhexine, has reduced the mortality of COVID-19 patients in clinical trials and is a potential therapeutic option as an inexpensive and safe over-the-counter drug [104]. In addition, the recently identified small molecule inhibitor of TMPRSS2, N-0385, inhibited SARS-CoV-2 infection in human lung cells and showed the high efficiency of prophylactic and therapeutic benefits in the transgenic mouse model of severe COVID-19 [105].

### 5.3. Immunoregulation Agents

The activation of immune cells in cytokine storms caused by SARS-CoV-2 infection increases the risk of death in severe patients. Targeting inflammation-relative signaling pathway proteins is also an important strategy for the treatment of COVID-19.

The JAK/STAT signaling pathway is activated in response to SARS-CoV-2 infection, which triggers inflammation, leads to cell recruitment and progression to cytokine storm, and produces various inflammatory markers in the host, and thus, determines disease severity [106]. By inhibiting JAK1/JAK2 selectively and effectively, baricitinib blocks the pro-inflammatory signals, and the immune cascade reduces the inhibitory effect of pro-inflammatory mediators on ENaC expression and alleviates lung injury [107]. Meanwhile, the two key regulators of the ACE2 receptor, AP-2-associated protein kinase 1 and cyclin G-associated kinase, can be bound by high-affinity baricitinib, which can block the invasion and replication of the virus. In clinical trials, baricitinib combined with standard treatment resulted in reduced mortality in hospitalized patients with COVID-19 [108,109]. Other JAK inhibitors, such as ruxolitinib, nezucitinib, and tolvastatin, also showed benefits in the treatment of COVID-19 patients [110].

Nuclear factor kappa B (NF-κB), a pro-inflammatory transcription factor, has been widely observed to be upregulated in the development of SARS-CoV-2 infection and has been considered a potential immunoregulation target for COVID-19 treatment [111]. Sharma et al. reported that curcumin could effectively inhibit the inflammatory response caused by the SARS-CoV-2 spike protein through inactivating MAPK/NF-κB signaling, which is characterized by reducing the expression levels of NLRP3, IL-1β, IL-18, and caspase-1, and inhibiting the inflammasome [112,113]. It has recently been shown that curcumin may restore the normal expression of fibrinolytic components, uPA and uPAR, and enhance the protein expression of ENaC, which may be used as a therapeutic agent in human pneumonia and ALI/ARDS caused by SARS-CoV-2 infection [114,115].

## 6. Conclusions

In this review, we targeted ENaC and plasmin for the characteristic pathological changes of ARDS and alveolar capillary microthrombus under SARS-CoV-2 infection. Based on the fact that the spike protein of SARS-CoV-2 shares the same furin site with ENaC and both can be activated by plasmin, the cleavage mechanism of SARS-CoV-2 is closely related to ENaC as considered. Viruses may hijack the cleavage mechanism of plasmin for ENaC during their invasion to host cells. When SARS-CoV-2 causes acute inflammatory responses in the lung, inflammatory factors are released, which increases PAI-1 and decreases the uPA/uPAR complex. In addition, the degradation of plasminogen fragments leads to a decrease in plasmin, which downregulates the open probability of ENaC channels. The internalized degradation, subcellular localization, and probability of channel opening under viral infection, as well as related mechanisms, clarify the possible intrinsic relationship among SARS-CoV-2, ENaC, and plasmin and provide a novel reference for clinical intervention in the treatment of ARDS. However, it is still unclear what is the superiority of SARS-CoV-2 in competing with ENaC for plasmin and what exactly is the role of plasmin among the many proteases that can interact with the furin site in vivo. These will need to be followed up for further understanding.

## Data Availability

The data presented in this study are available on request from the corresponding author.
